# Challenges hindering memristive neuromorphic hardware from going mainstream

**DOI:** 10.1038/s41467-018-07565-4

**Published:** 2018-12-10

**Authors:** Gina C. Adam, Ali Khiat, Themis Prodromakis

**Affiliations:** 1 Department of Electrical and Computer Engineering, George Washington University, Washington, DC 20052 USA; 2 Electronic Materials & Devices, Zepler Institute for Photonics and Nanoelectronics, University of Southampton, Southampton, SO17 1BJ, UK

## Abstract

Memristive devices have elicited intense research in the past decade thanks to their inherent low voltage operation, multi-bit storage and cost-effective manufacturability. Nonetheless, several outstanding performance and manufacturability challenges have prevented the widespread industry adoption of redox-based memristive matrices. Here, we discuss these challenges in terms of key metrics and propose a roadmap towards realizing competitive memristive-based neuromorphic processing systems.

## The promise of redox memristors

Heterogeneous hardware that combines traditional digital circuitry with two-terminal analog memory devices, promises to handle the Zettabyte storage and processing requirements of modern applications such as the Internet of Things (IoT) and Artificial Intelligence (AI). Several emerging device concepts, based on electrochemical metallization, phase change, and redox phenomena have been intensely explored. This work led to some successful commercial products, like Adesto’s Moneta electrochemical metallization memory for low-energy applications and Intel-Micron’s phase change memory Optane for storage class memory. Despite this fact, the most highly sought application nowadays, namely non-volatile neuromorphic processors, has yet to become industrially feasible.

We believe that redox memristive memory will be the technology to fuel the AI era in the upcoming decades by enabling competitive implementations of neuromorphic processors. These switches can facilitate the energy and space efficiency required for emulating synaptic weights—the programmable connections that equip a neuromorphic system with its learning and memory capabilities. The synaptic weights can be implemented with commercially available technologies, but they typically require tens of devices for emulating a single synapse, which renders large-scale systems impractical. For comparison, redox memristive cells can outshine by 2–3 orders of magnitude in density and lower energy consumption of the implementations featuring more mature technologies^1^. To emulate the complexity and ultra-low power consumption of biological neural networks, neuromorphic hardware platforms have to deliver an ultra-high density (>1 Tb/cm^2^) and energy efficient (<10 fJ/operation) solution. If we want to implement large neural networks with billions of synaptic devices, resistive switches are particularly suited thanks to three disruptive attributes: low-voltage multi-bit programmability, an inherent non-volatility of their resistance state, and a scalable two-terminal structure appropriate for matrix integration.

The physics of resistive switching is on our side from an energy-consumption perspective, since in theory the state of the device can change through the movement of just a few ions under a very low voltage. Once the voltage is removed, the ions halt in place and the state is retained without any further use of energy. The fine synaptic programmability is a key element for neuromorphic algorithms and redox resistive devices have achieved the best analog capacity to date (>100 discernible states per single cell)^2^. Redox resistive devices are bipolar so a desired state can be accessed either during set or reset, which decreases the latency to program the matrix. Redox memristors typically report the lowest energy consumption/switching among emerging analog memory solutions, ~10fJ^3^. Moreover, the switching time has been shown to be as low as 85 ps^4^ for nitride materials.

An ideal neuromorphic platform would take advantage of these properties in an integrated fashion. Such a system would have hundreds of layers of resistive switching matrices integrated over traditional digital circuitry to achieve high performance at a low manufacturing cost.

## Performance vs manufacturability challenges

This bold dream has fueled intense research in the field. Significant progress has been made, but in all honesty, at a slower pace than anticipated. No miracle material stack that leads to the perfect device properties has been discovered yet. Several performance and manufacturability challenges prevent industry adoption. Yet we are optimistic that our community will overcome these challenges and develop a resistive switching technology of unparalleled performance for the next generation of neuromorphic hardware.

### Variability

While neuromorphic computation is considered to be resilient to hardware defects, memristor variability is costly. If each device performs slightly different and its characteristics vary in time, programming to a desired state becomes a personalized endeavor. This approach is not feasible for training large matrices with billions of devices, as it consumes time, energy, and chip real-estate for supporting circuitry.

**Fig. 1:**
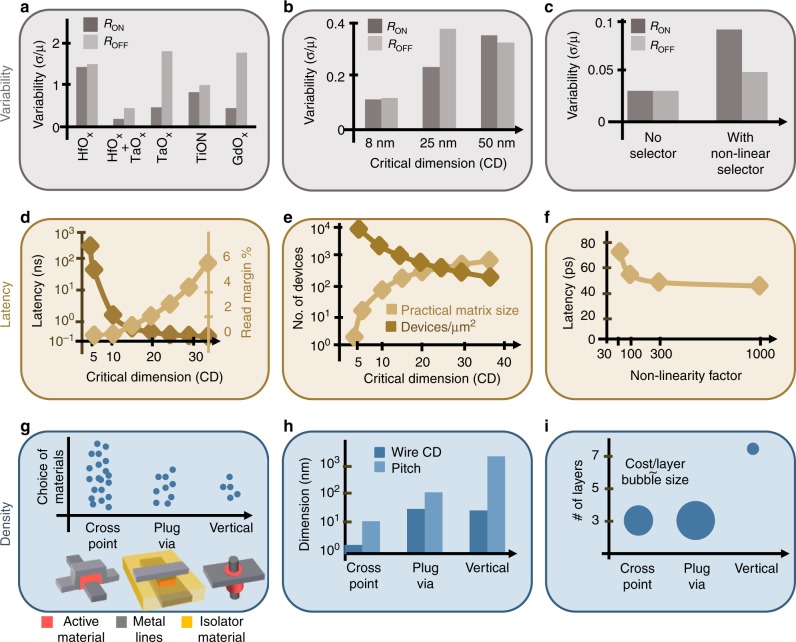
Matrix-level metrics and manufacturing choices impacting them. **a**–**c** Variability metric. The variability is a measure of the spread of device performance (in this example, the two extreme resistance states *R*_ON_ and *R*_OFF_) in a memristive matrix as defined based on the standard deviation and the means of the resistance distributions (*σ*/*μ*). The variability of the resistance states *R*_ON_ and *R*_OFF_ across a matrix is heavily influenced by **a** the choice of active material and of the material stack (e.g., single material HfO_x_ vs. bilayer HfO_x_ + TaO_x_)^5, 12^; **b** the device scaling as determined by the smallest feature dimension (also known as critical dimension or CD);^6^ and **c** the presence of a series selector/cell which has its own variability profile^8^. The variability results presented in **a**–**c** are extracted from different studies so they have different orders of magnitude depending on the manufacturing process used. **d**–**f** Latency metric. The latency is a measure of the delay in accessing the desired device, delay caused by the charging and discharging of the wires. **d** Impact of the wire downscaling on latency and read margin, which is a measure of the capability to discriminate between the two extreme states (*R*_ON_ and *R*_OFF_) of the memristive device^13^. **e** Practical matrix size limited by latency vs. the density (number of devices in a μm^2^) allowed by the critical dimension of the manufacturing process. **f** The impact of the device / selector non-linearity on latency^14^. **g**–**i** Density metric discussed from the perspective of the most common device designs—crosspoint, plug-via and vertical. **g** The availability of materials suitable for each device design, given aspects such as uniformity, conformal deposition, etc. **h** The state-of-the-art scalability for each design (crosspoint: 2 nm CD/12 nm pitch^9^, plug-via ~30 nm/100 nm^15^, vertical structure has yet to be optimized for scalability^12^). **i** State-of-the-art stackability for each design and its approximate cost per matrix layer (represented by the relative size of the bubble)

High-density integration and mass production will not be possible until the variability is fixed. And fixing it is challenging. This is a new technology that requires significant investment for refining the design and manufacturing process. More alarming is, however, the intrinsic stochastic nature of the switching. The resistive switching technology has been extensively shown in amorphous or polycrystalline materials. These materials have the advantage of low temperature deposition, so multiple matrix layers can be manufactured without disturbing the digital circuitry below. However, their uncontrolled high density of defects induces a high degree of variability. The choice of materials plays a critical role^5^ (Fig. 1a). Extreme scaling has also been shown to reduce variability, probably through confining the area where switching occurs^6^ (Fig. 1b). In the meantime, more complex cells, like the multi-memristor cell used to emulate a single synaptic unit^7^, can help alleviate some of these challenges, but at the cost of lower integration density.

### Latency

While variability limits the size of the system that we can build, this is not our only challenge. The practical size of the matrix is limited also by the accessibility of individual devices in the matrix. The line resistance can determine a non-negligible voltage drop across the wires, increasing the latency (the time it takes to access a device) and the energy consumption and affecting the write/read margin (Fig. 1d). Sneak paths are another issue that aggravates with increased matrix size. A highly non-linear selecting device (called selector) in series with each memristor offers increased accessibility, as higher nonlinearity is desirable for reduced latency (Fig. 1f). Nevertheless, selectors have their own variability that further adds to the deterioration of performance^8^ (Fig. 1c). These issues become more acute with drastic technology scaling and limit the realistic matrix size (Fig. 1e).

### Density

Despite the abovementioned limitations, the promise for an extremely small footprint provides a clear advantage by comparison with more mature technologies like flash memory. Various designs can be used, with the crosspoint, plug-via and vertical topologies being the most explored. Each has its merits and challenges, requiring trade-offs in scalability, stackability, selector integration capabilities and cost effectiveness. The crosspoint is the most common, due to easy manufacturing with a wide range of materials (Fig. 1g) and its extreme scalability, down to ~2 nm for an estimated density of >0.7Tb/cm^2^ ^9^ (Fig. 1h). However, it has the major disadvantage of the active material stepping over the bottom line which can cause uncontrolled film thinning, increased device variability, or even electrodes shorting. The plug-via design has no step, but needs the etching of the via which damages the active film, increases the variability and requires additional masks. The vertical design is, by comparison, highly cost effective (Fig. 1i). The number of masks is independent of the number of layers, similar to the three-dimensional flash technology^10^. However, the requirement for conformal vertical deposition limits the choice of materials and of selector integration.

While the quest for the densest matrix design is admirable, a memristor-based neuromorphic processor is more than memristor matrices. Additional circuitry is typically required for selection, reading and programming of cells. Ideally, this circuitry would be implemented entirely below the memristor matrix stack for attaining highest chip space occupancy. However, high speed programming requirements can increase the circuitry footprint, thus straying away from the ideal density^11^.

## Reaching technological feasibility

**Fig. 2:**
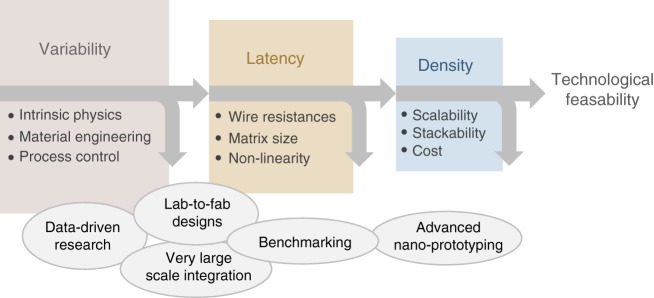
Roadmap for manufacturing challenges and possible approaches to accelerate progress. Understanding the underlying factors behind variability can be enabled by data-driven research through lab-to-fab designs and very large-scale integration of memristive matrices with traditional digital access circuitry. Benchmarking (performance metrics, standardized device/matrix sizes, methods of testing, etc) will ensure comparable results between groups. Ultimately, once variability and latency issues are tackled, the technology development will benefit from advanced nano-prototyping techniques, such as extreme ultraviolet lithography, for cost-effective scalability and stackability

Driven by its potential for extreme density, resistive switching matrices will benefit from the latest advances in nanofabrication, like the extreme ultraviolet lithography (EUV) which has already shown <10 nm half pitch lines. However, the industry can benefit from its technological potential only when the issues of variability and latency are solved, so that should be the short-term focus in our opinion (Fig. 2).

Tackling them requires a data-driven approach to accelerate the understanding and gaining control over the physics of switching, the materials and the manufacturing process. The necessity of having low access resistance and selector devices introduces extra complexity, requiring designs with higher number of manufacturing steps and state-of-the-art cleanroom equipment. The characterization of large matrices is resource intensive as well, involving custom data acquisition set-ups. The solution is the integration of memristor matrices with the digital read/write circuitry which requires foundry material compatibility and sustained academia-industrial partnerships. Appropriate performance benchmarking amongst distinct materials, standardized device/matrix sizes and methods of testing are also needed to ensure reproducible results across different labs. A repository of these large datasets would strengthen the research capabilities of the community, enabling accurate device modeling and system-level simulations.

In the coming years, memristive neuromorphic hardware will likely flourish in select embedded applications based on medium-sized matrices suitable for cost-effective training off-site and pre-deployment. Complex systems would take longer to reach commercial maturity since they require larger memristive matrices with lower density of imperfections appropriate for fast on-site continuous learning. Ultimately though, the balance between system-level performance vs. manufacturing cost will be what drives widespread adoption.
